# Supplementary material for: Identification of photoperiod-induced specific miRNAs in the adrenal glands of Sunite sheep (Ovis aries)
Source: Front Vet Sci. 2022 Jul 22;9:888207. doi: 10.3389/fvets.2022.888207 (PMC9354845; doi:10.3389/fvets.2022.888207)
Supplement: Supplementary file 1 [file Data_Sheet_1.ZIP › Supplementary file/miRDeep2 file.zip!/miRDeep2 file/Novel_Structure/SPLP42A2/Novel_422.pdf]

Provisional ID	:	NC_019460.2_9296
Score total	:	2.6
Score for star read(s)	:	-1.3
Score for read counts	:	0
Score for mfe	:	2.3
Score for randfold	:	1.6
Score for cons. seed	:	
Total read count	:	67
Mature read count	:	67
Loop read count	:	0
Star read count	:	0

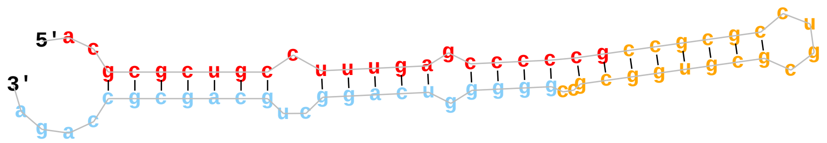

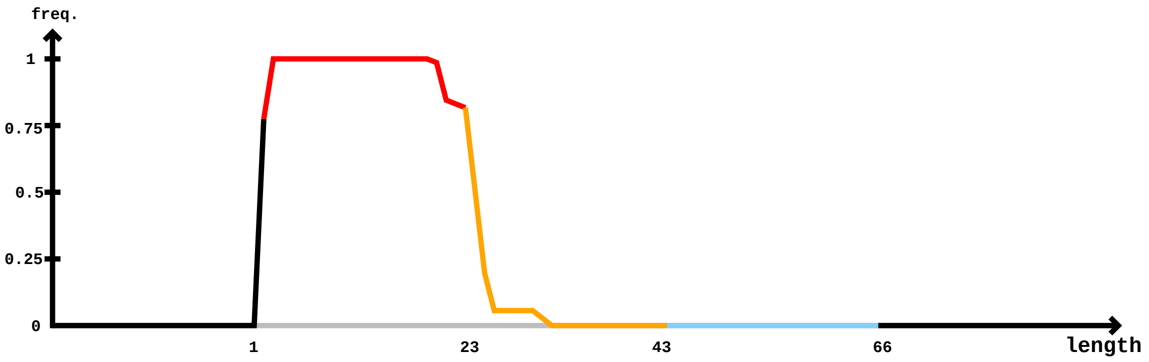


```
                     Mature                                          Star
5'- ccaaucuaaaugucugaugaacgcgcugccuuugagcccccgccgcgccugcgcguggcgccgggggucaggcugcagcgccagaggacaugcucgcgcauaacugggg  -3'   exp
    (((......((((((..((...(((((((.(((((.((((((((((((....))))))))..)))).))))).)))))))))..))))))((....)).....)))..   reads      mm         sample
    ....................acgcgcugccuuugagcc.......................................................................   1          0          seq
    ....................acgcgcugccuuugagccc......................................................................   8          0          seq
    ....................acgcgcugccuuugagcccc.....................................................................   1          0          seq
    ....................acgcgcugccuuugagcccccg...................................................................   29         0          seq
    ....................acgcgcugccuuugagcccccgc..................................................................   8          0          seq
    ....................acgcgcugccuuugagcccccgcc.................................................................   4          0          seq
    ....................acgcgcugccuuugagcccccgccgcgcc............................................................   2          0          seq
    ....................acgcgcugccuuugagcccccgccgcgccu...........................................................   2          0          seq
    .....................cgcgcugccuuugagccc......................................................................   2          0          seq
    .....................cgcgcugccuuugagccccc....................................................................   1          0          seq
    .....................cgcgcugccuuugagcccccg...................................................................   6          0          seq
    .....................cgcgcugccuuugagcccccgc..................................................................   1          0          seq
    .....................cgcgcugccuuugagcccccgcc.................................................................   6          0          seq
```
